# Perturbation of formate pathway and NADH pathway acting on the biohydrogen production

**DOI:** 10.1038/s41598-017-10191-7

**Published:** 2017-08-29

**Authors:** Dong Liu, Yunze Sun, Yuhao Li, Yuan Lu

**Affiliations:** 1 Department of Chemical Engineering, Tsinghua University, Beijing, 100084 China; 2 Key Lab of Industrial Biocatalysis, Ministry of Education, Department of Chemical Engineering, Tsinghua University, Beijing, 100084 China; 3 Institute of Biochemical Engineering, Department of Chemical Engineering, Tsinghua University, Beijing, 100084 China

## Abstract

The formate pathway and NADH pathway as two common hydrogen-producing metabolic pathways have been well characterized to understand and improve biohydrogen production. These two pathways have been thought to be separate and have been independently investigated. However, in this study, perturbation of genes (*hycA*, *fdhF*, *fhlA*, *ldhA*, *nuoB*, *hybO*, *fdh1*, *narP*, and *ppk*) in *Enterobacter aerogenes* related to the formate pathway or NADH pathway revealed that these two pathways affected each other. Further metabolic analysis suggested that a linear relationship existed between the relative change of hydrogen yield in the formate pathway or NADH pathway and the relative change of NADH yield or ATP yield. Thus, this finding provides new insight into the role of cellular reducing power and energy level in the hydrogen metabolism. It also establishes a rationale for improving hydrogen production from a global perspective.

## Introduction

Fossil fuels have been the conventional and principal sources to satisfy the world’s demand of energy. However, their intensive use has caused environmental problems^1^. In such a context, hydrogen can be a promising future energy carrier, which is pollution free for its combustion only generating water without any byproduct^2^. At present, 40% of hydrogen is produced from natural gases, 30% from heavy oil and naphtha, 18% from coal, and 4% from electrolysis^3^. However, these production methods require highly intensive energy sources. It is worthless to produce clean energy by consuming much energy and still cause environmental problems. In the present scenario, biohydrogen production is of great importance to be an alternative^2, 4^.

Compared to conventional production methods, biological hydrogen production can use renewable resources as the reactant and be operated at ambient temperature and pressure, which means much less energy input and lower cost^4^. There are three basic production methods: direct biophotolysis, photofermentation, and dark fermentation. Compared to the first two methods, dark fermentation seems to be the most practical and promising method on account of having no need for direct solar energy input, while having a wide substrate range from energy crops to waste streams, and requiring only simple reactor technology. However, the dark fermentation method is currently limited by low hydrogen yield^5, 6^. Therefore, many studies focus on how to improve the hydrogen yield of dark fermentation by metabolic engineering.

**Figure 1:**
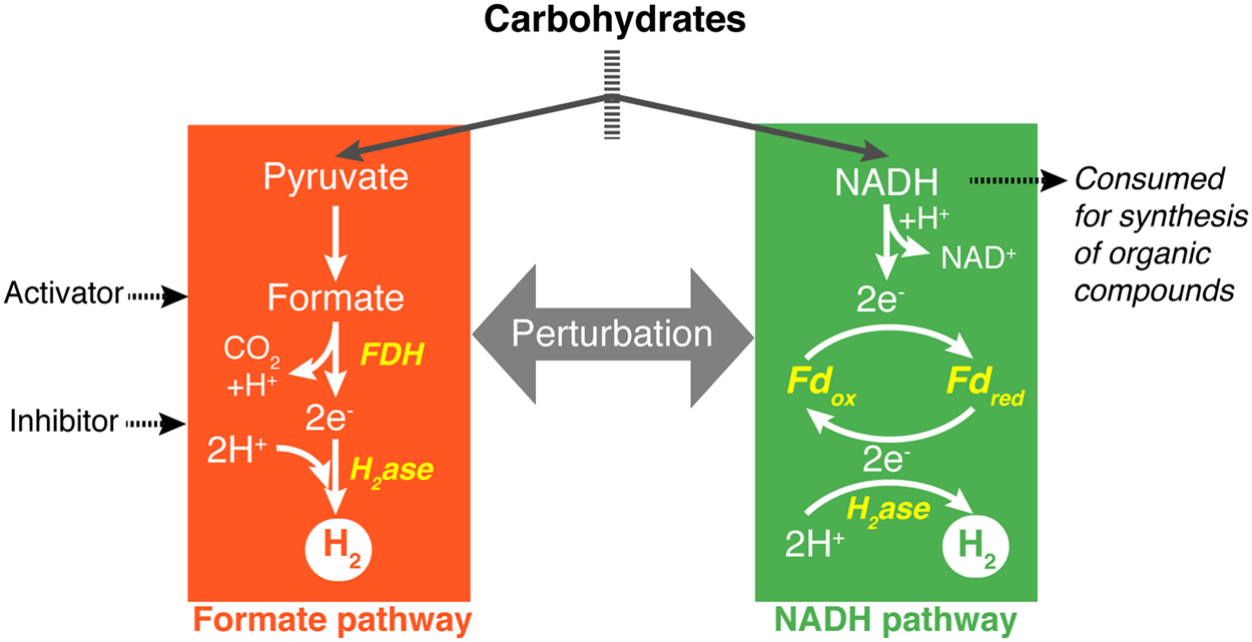
Two main hydrogen-producing pathways in dark fermentation: formate pathway and NADH pathway.

To improve the hydrogen yields, many attempts have been made to understand the hydrogen producing mechanism of dark fermentation in depth. Various microorganisms, typically the anaerobes, have been used for dark fermentation, such as *Escherichia coli*, *Clostridium* sp.^7^, *Enterobacter* sp.^8^, and *Rhodobacter* sp.^9^. Although each strain has its different metabolic features, there are two main hydrogen-producing pathways in common: the formate pathway and NADH pathway (Fig. 1). To enhance hydrogen yields, many attempts focusing on the formate pathway or the NADH pathway have been made to redistribute the metabolic fluxes by genetic engineering.

The key element of the formate pathway is the formate hydrogen lyase (FHL) complex^10^. The FHL catalyzes the formate oxidation and the proton reduction, and ultimately produces molecular hydrogen and carbon dioxide at a 1:1 molar ratio. The core FHL complex comprises of formate dehydrogenase (FDH) and hydrogenase (H_2_ase) (Fig. 1). Many genetic engineering studies about the FHL-related genes have been done to regulate the metabolism of the formate pathway to enhance the hydrogen production^10–13^. The NADH pathway was first reported in the 1980s^7^, with NADH as the precursor involved in the hydrogen production through ferredoxin(Fd)-NAD^+^ reductase and ferredoxin hydrogenase. Many steps have been made to regulate the metabolism of NADH to enhance the H_2_ production^7, 14–16^.

The formate pathway and NADH pathway have been thought to be independent, but recent studies demonstrated that there might be a link between them (Fig. 1). In Niu’s research on *Klebsiella pneumoniae*, a higher temperature can enhance the hydrogen-producing NADH pathway, while the flux in the formate pathway reach their maximum at pH 7.0–7.5^17^. Ma *et al*. found that the hydrogen production fluxes in the formate pathway and the NADH pathway both increased when NADH dehydrogenase was impaired in *Enterobacter aerogenes* ^18^. It seems that regulation of the NADH pathway is accompanied by the perturbation of the formate pathway.

To figure out the puzzle of the interaction between the formate pathway and the NADH pathway, further research on metabolic regulation of fermentative hydrogen production is needed. In this study, a representative hydrogen-producing bacterium, facultatively anaerobic *E. aerogenes*, was used. Two hydrogen-producing pathways, the formate pathway and the NADH pathway, coexist in *E. aerogenes*. Genes related to the formate pathway or NADH pathway were perturbed, and then the hydrogen production was investigated. Cellular energy metabolism was further explored by metabolic analysis. Findings obtained in this study could help to thoroughly understand how perturbation of formate pathway and NADH pathway act on the hydrogen metabolism.

## Results

### Perturbation of the formate pathway and NADH pathway in wild-type *E. aerogenes*

**Figure 2:**
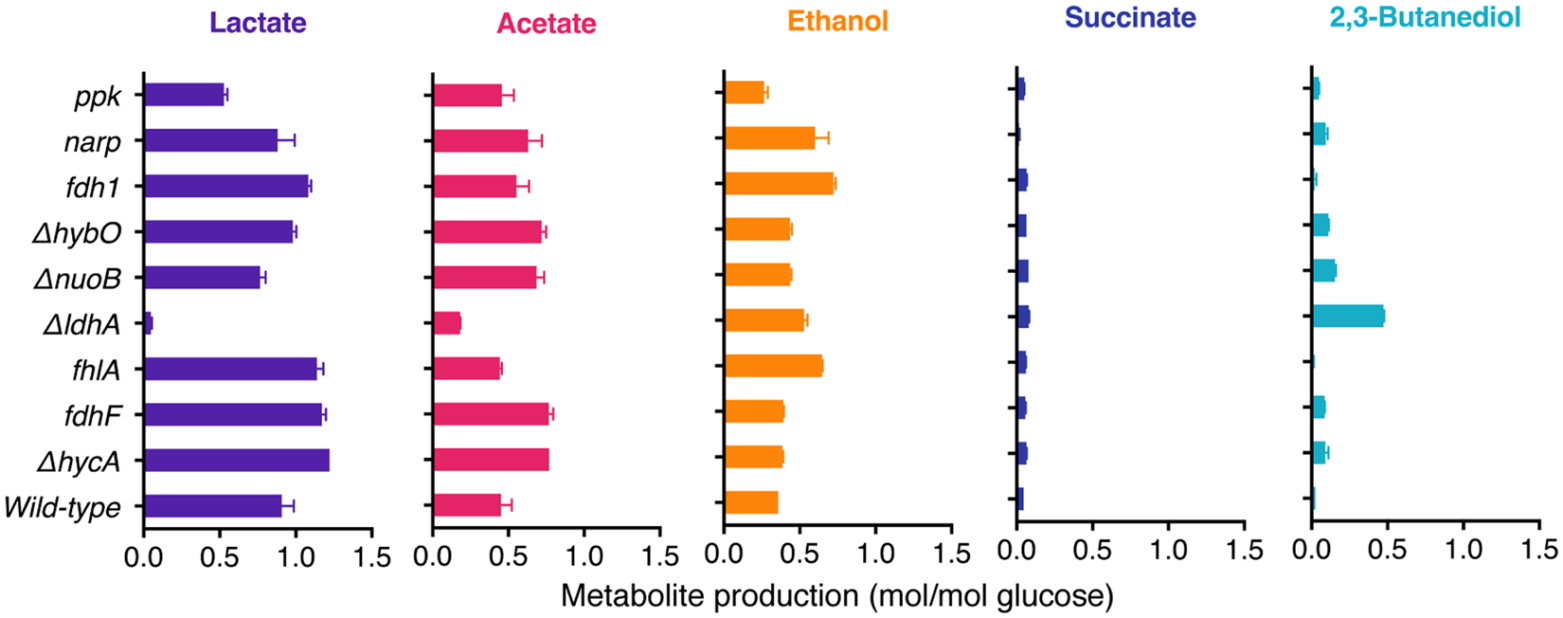
Metabolite analysis of *E. aerogenes* mutants with different single gene knockout or overexpression.

To explore the perturbation of the formate pathway and NADH pathway in wild-type *E. aerogenes*, a fourfold strategy was adopted (Table 1). First, mutations directly related to the formate pathway were evaluated. In the wild-type strain, knockout of *hycA* (FHL repressive regulon gene), overexpression of *fdhF* (formate dehydrogenase H gene), or overexpression of *fhlA* (FHL activator protein FHLA gene) made the hydrogen yields increase from the formate pathway but decrease from the NADH pathway. Second, mutations directly related to the NADH pathway were then evaluated. The most NADH-consuming pathway is lactate production pathway. The knockout of *ldhA* (lactate dehydrogenase gene) greatly improved the hydrogen yield from the NADH pathway, but the hydrogen yield from the formate pathway had no change. The *nuoB* gene encoding the NADH dehydrogenase/NADH-quinone oxidoreductase was impaired, which made the hydrogen yields from the formate pathway and NADH pathway both increase. The third task was directly altering both the formate pathway and NADH pathway at the same time. Knockout of *hybO* (uptake hydrogenase gene) improved the hydrogen yields from both the formate pathway and NADH pathway. An enzyme called NAD^+^-dependent formate dehydrogenase can work on both formate and NADH, which uses formate to regenerate NADH from NAD^+^^19^. The gene *fdh1* encoding NAD^+^-dependent formate dehydrogenase from *Candida boidinii* was expressed into wild-type *E. aerogenes*. It resulted in the increase of hydrogen yields from the formate pathway but the decrease of hydrogen yields from the NADH pathway. The fourth strategy was alternating the whole cellular metabolism that might indirectly affect the hydrogen production. A reported global regulator NarP encoded by *narP* is a transcriptional regulator of many anaerobic electron transport and fermentation-related genes^20^. The polyphosphate kinase (PPK) encoded by *ppk* regulates cellular energy level^21, 22^. Overexpression of *narP* or *ppk* improved the hydrogen yields from both formate pathway and NADH pathway. The relative change of hydrogen yield in the NADH pathway was bigger than that in the formate pathway. Further metabolite analysis (Fig. 2) showed that cellular metabolism was greatly regulated. The knockout of *ldhA* changed the metabolism the most.

**Table 1:** Relative change of hydrogen yields by *E. aerogenes* mutants with different single gene knockout or overexpression.

Knockout^a^	Overexpression^b^	Relative change of H_2_ yield^c^ (%)		
		Total^d^	Formate pathway^e^	NADH pathway^f^
Genes directly related to formate pathway				
*hycA*	—	11.5 ± 0.19	20.3 ± 0.32	−11.5 ± 2.40
—	*fdhF*	15.7 ± 0.45	20.7 ± 0.23	2.40 ± 0.03
—	*fhlA*	4.60 ± 0.16	13.8 ± 0.77	−18.9 ± 1.45
Genes directly related to NADH pathway				
*ldhA*	—	12.4 ± 0.38	0.03 ± 0.01	39.7 ± 0.42
*nuoB*	—	22.3 ± 1.32	16.8 ± 0.26	36.3 ± 1.66
Genes directly related to both formate pathway and NADH pathway				
*hybO*	—	22.2 ± 0.68	19.3 ± 0.46	29.3 ± 0.80
—	*fdh1*	11.5 ± 0.70	32.7 ± 0.66	−42.9 ± 2.57
Genes indirectly related to both formate pathway and NADH pathway				
—	*narp*	18.2 ± 2.45	9.10 ± 1.37	45.9 ± 6.42
—	*ppk*	32.7 ± 3.35	13.2 ± 1.09	77.7 ± 4.86

The genes for knockout or overexpression were directly or indirectly related to the formate pathway or NADH pathway. Each data point indicates the mean of triplicate assay results.

^a^The genes *hycA*, *ldhA*, *nuoB* and *hybO* encode formate hydrogen lyase repressive regulon, lactate dehydrogenase A chain, NADH dehydrogenase/NADH-quinone oxidoreductase, and uptake hydrogenase.

^b^The genes *fdhF*, *fhlA*, *fdh1*, *narP* and *ppk* encode formate dehydrogenase H, formate hydrogen lyase activator, NAD^+^-dependent formate dehydrogenase from *Candida boidinii*, global regulator NarP, and polyphosphate kinase.

^c^Relative change of H_2_ yield = (H_2_ yield of the mutant - H_2_ yield of the blank control)/H_2_ yield of the blank control.

^d^Total H_2_ yield was obtained based on the measurements of hydrogen production and glucose consumption, which was equal to sum of hydrogen yields produced from the respective formate pathway and NADH pathway.

^e^H_2_ yield produced by formate pathway = (acetate + ethanol-formate)/consumed glucose.

^f^H_2_ yield produced by NADH pathway was calculated the sum of pathways producing NADH minus those consuming NADH (H_2_ produced by NADH pathway = ((2 × glucose + CO_2_ + CO_2_ consumed by succinate production-2 × 2,3-butanediol)-(2 × ethanol + 2 × succinate + lactate + 2,3-butanediol + H_2_-CO_2_))/consumed glucose).

### Perturbation of the formate pathway and NADH pathway in *E. aerogenes* mutants

**Figure 3:**
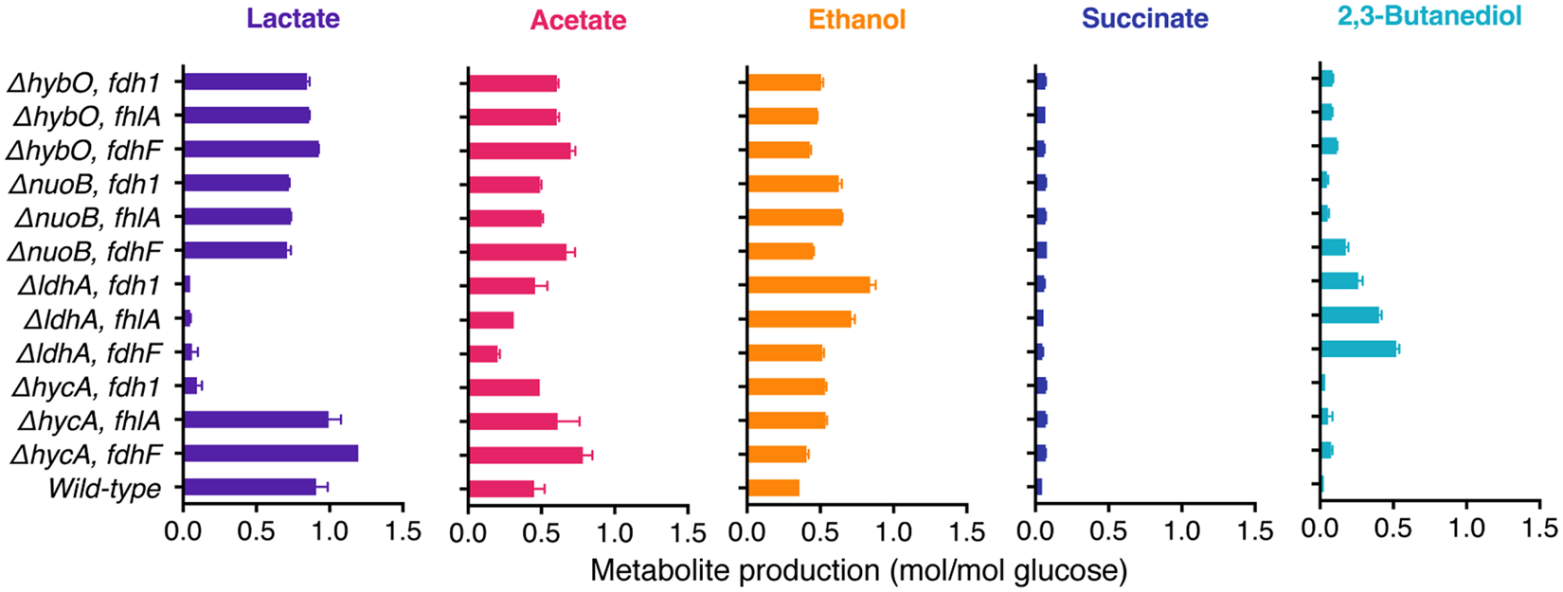
Metabolite analysis of strains with the overexpression of different genes in different *E. aerogenes* mutants.

Furthermore, in different *E. aerogenes* mutants, *fdhF*, *fhlA* or *fdh1* were overexpressed to investigate the perturbation of the formate pathway and NADH pathway (Table 2). In the *hycA*-deficient strain, overexpression of *fdhF*, *fhlA* or *fdh1* greatly disturbed the hydrogen production in the NADH pathway. However, in *ldhA*-deficient strain, overexpression of *fhlA* or *fdh1* greatly disturbed the hydrogen production in the formate pathway. In contrast,, in the *nuoB*-deficient or *hybO*-deficient strain, overexpression of *fdhF*, *fhlA* or *fdh1* almost did not affect the hydrogen production in the formate pathway or the NADH pathway. Besides hydrogen, other key cellular metabolites were measured (Fig. 3). In the *ldhA*-deficient strain, overexpression of *fdhF*, *fhlA* or *fdh1* affected cellular metabolism more obviously than other gene perturbations. Overexpression of *fdhF* regulated cellular levels of lactate, acetate, and 2,3-butanediol better than the overexpression of *fhlA* or *fdh1*. Overexpression of *fdh1* regulated cellular ethanol level better than overexpression of *fdhF* or *fhlA*.

**Table 2:** Relative change of hydrogen yields after the overexpression of different genes in different *E. aerogenes* mutants.

Mutants	Overexpression	Relative change of H_2_ yield (%)		
		Total	Formate pathway	NADH pathway
*hycA* deficient	*fdhF*	6.86 ± 0.25	2.86 ± 0.10	20.8 ± 0.98
	*fhlA*	7.94 ± 1.16	−0.69 ± 0.02	38.3 ± 2.67
	*fdh1*	−2.62 ± 0.22	−11.5 ± 0.21	28.3 ± 0.49
*ldhA* deficient	*fdhF*	−0.70 ± 0.15	1.28 ± 0.04	−3.90 ± 0.13
	*fhlA*	26.8 ± 0.30	45.1 ± 1.35	−2.75 ± 0.09
	*fdh1*	49.2 ± 8.18	84.3 ± 3.74	−7.57 ± 0.45
*nuoB* deficient	*fdhF*	−0.74 ± 0.04	0.27 ± 0.01	−2.94 ± 0.09
	*fhlA*	0.74 ± 0.01	2.77 ± 0.02	−3.52 ± 0.06
	*fdh1*	1.41 ± 0.02	−0.27 ± 0.01	5.09 ± 0.21
*hybO* deficient	*fdhF*	−4.66 ± 0.09	−1.49 ± 0.03	−12.2 ± 0.30
	*fhlA*	−3.37 ± 0.05	−5.24 ± 0.01	1.24 ± 0.01
	*fdh1*	−5.09 ± 0.10	−3.06 ± 0.09	−9.90 ± 0.30

The overexpressed genes were directly related to the formate pathway or NADH pathway.

### Quantitative relationship of hydrogen production with cellular energy metabolism

**Figure 4:**
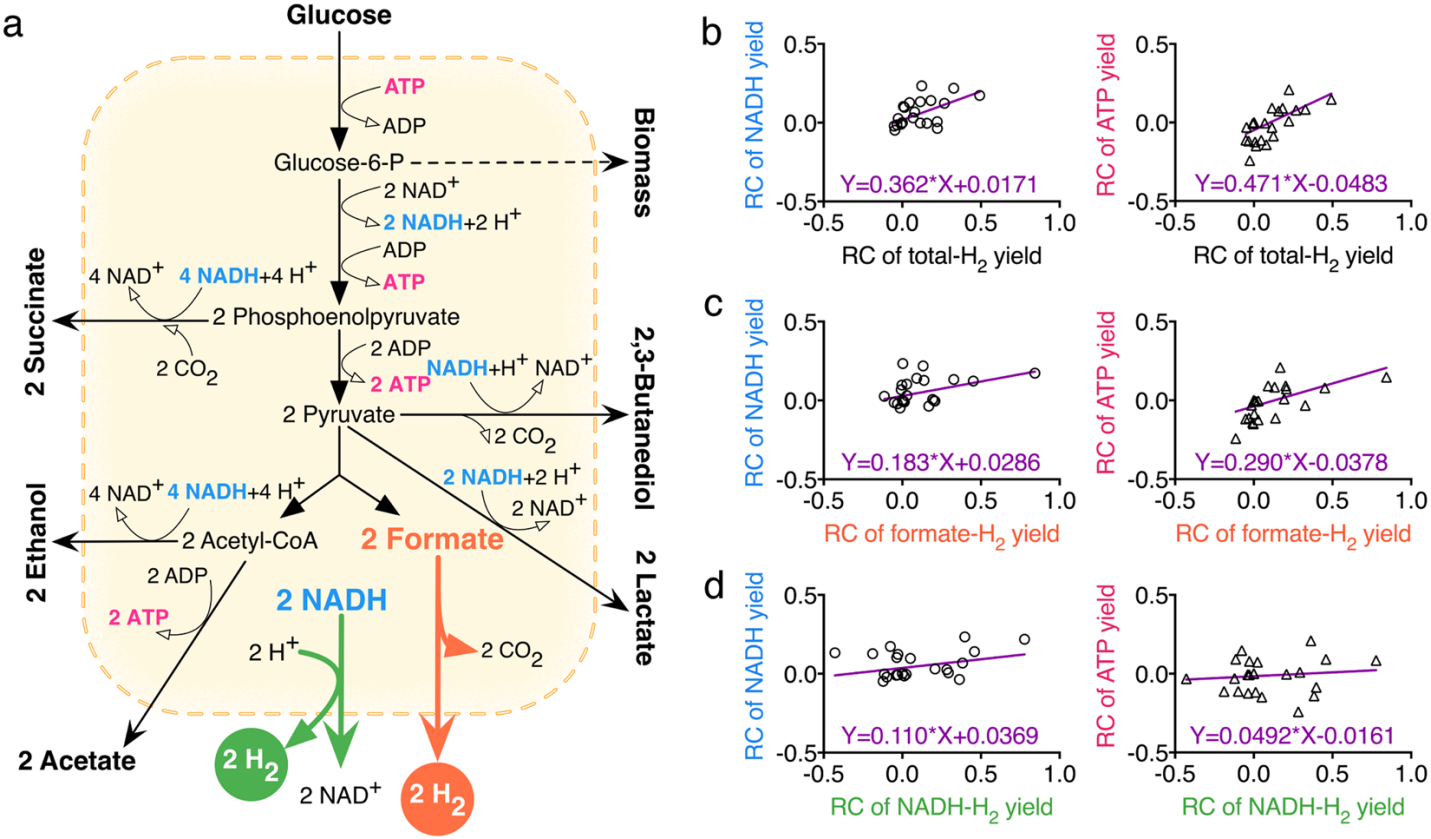
In cellular anaerobic metabolism (**a**), quantitative relationships between NADH yield or ATP yield and the total hydrogen yield (**b**), the hydrogen yield in the formate pathway (**c**), or the hydrogen yield in the NADH pathway (**d**). Hydrogen yield values were in moles of hydrogen produced per mol of consumed glucose. The NADH yield values were in moles of consumed NADH (2 × ethanol + 2 × succinate + lactate + butanediol + NADH for hydrogen production) per mol of consumed glucose. ATP yield values were in moles of ATP produced (lactate + acetate + formate + CO_2_ + CO_2_ consumed by succinate production) per mol of consumed glucose. Relative change (RC) of yield = (Yield after the overexpression of genes - Yield of the blank control)/Yield of the blank control.

Perturbation of the formate pathway and NADH pathway inevitably led to the change of the whole anaerobic metabolism. Besides H_2_ and CO_2_, other main final metabolites of *E. aerogenes* are lactate, ethanol, acetate, succinate, and 2,3-butanediol (Fig. 4a). Based on metabolic analysis, the relationships between hydrogen productivity and NADH level or ATP level were quantitatively evaluated. By perturbing genes (*hycA*, *fdhF*, *fhlA*, *ldhA*, *nuoB*, *hybO*, *fdh1*, *narP*, and *ppk*) related to the formate pathway or NADH pathway (Tables 1 and 2), cellular metabolites (Fig. 4a) were measured and analyzed to evaluate the change of cellular NADH and ATP level. Metabolic analysis suggested that there were linear relationships between the relative change of hydrogen yields (total yields, yields in the formate pathway, or yields in the NADH pathway) and the relative change of NADH yields or ATP yields (Fig. 4b–d).

## Discussion

To study the perturbation of the formate pathway and NADH pathway acting on the hydrogen metabolism, choosing a proper model strain is particularly important. Among the fermentative hydrogen producers, the facultative anaerobe *E. aerogenes* with excellent hydrogen-producing ability can be applicable for this work. Two hydrogen-producing pathways, the formate pathway and the NADH pathway, coexist in *E. aerogenes*, which provides a stable and balanced cellular environment for these two pathways. Therefore, investigating the interaction of both the formate pathway and NADH pathway can be quite convenient and easy in *E. aerogenes*. Furthermore, *E. aerogenes* shows a high hydrogen-producing rate and a high growth rate with no inhibited growth in 100% H_2_ atmosphere. A wide range of carbon sources can be used by *E. aerogenes*, which implies the great potential for industrial applications^23, 24^.

In the formate pathway, the structure and the mechanism of the FHL at the gene level is already clear, such as the sequence of its operons and regulons. Therefore, many genetic engineering studies about the FHL-related genes have been done to regulate the metabolism of the formate pathway to enhance the hydrogen production (Supplementary Table S2). These operations include inactivation of *hycA* (a negative regulator)^25^ and *focA* (formate transmembrane protein)^26^, as well as overexpression of *fhlA* (a transcriptional activator)^27, 28^, *fdhF* (formate dehydrogenase H)^29^, *modE* (a transcriptional activator)^26^ and *selC* (tRNA related to formate dehydrogenase)^26^.

The regulation of the NADH pathway is different from the formate pathway. Since the metabolic mechanism and genetic information of the NADH pathway are unclear, it is difficult to regulate the expression of enzymes on the NADH pathway. Fortunately, NADH participates in a wide range of metabolic reactions, in which the NAD(H) pool keeps a dynamic balance. Therefore, many steps have been made to regulate the metabolism of NADH to enhance the H_2_ production (Supplementary Table S2), for example, usually reducing the consumption of NADH of other metabolic pathways. In anaerobic metabolism, NADH generated from glycolysis is mainly consumed by the lactate production pathway and the alcohol production pathway, so the disruption of the lactate dehydrogenase gene *ldhA* and the alcohol dehydrogenase gene *adh* to increase H_2_ production is feasible^30^. Some other genes or gene clusters related to the NADH metabolism such as *frdBC* ^16^, *nuoB* ^29^, *nadE* ^15^ and *hoxEFUYH* ^31^ have also been regulated to improve the hydrogen yields.

In this work, genes (*hycA*, *fdhF*, *fhlA*, *ldhA*, *nuoB*, *hybO*, *fdh1*, *narP*, and *ppk*) directly or indirectly related to the formate pathway or NADH pathway were perturbed. The genes *hycA*, *fdhF* and *fhlA* are directly related to the formate pathway, and the genes *ldhA* and *nuoB* are directly related to the NADH pathway. Furthermore, the genes *hybO* and *fdh1* are directly related to both the formate pathway and NADH pathway. The genes *narP* and *ppk* alternate cellular global metabolism, which are indirectly related to hydrogen-producing pathways. The exploration of the perturbation of those genes further confirmed that these two pathways mutually impacted each other or were regulated together. The increased ratios of total hydrogen yield by the knockout of the *nuoB* gene or the *hybO* gene were much higher than that by the knockout of other genes, as shown in Table 1. In most case, as shown in Table 1, the NADH pathway for the hydrogen production was more easily perturbed than the formate pathway. Additionally, it is proposed that the increase of hydrogen yields might have a limit in *E. aerogenes*, which may be the reason why overexpression of *fdhF*, *fhlA* or *fdh1* in *nuoB*-deficient or *hybO*-deficient strains almost did not affect the hydrogen production, as shown in Table 2. Besides the hydrogen, analysis of other metabolites further demonstrated that cellular metabolism has been greatly regulated by perturbations of those genes (Figs 2 and 3). Main final organic metabolites of *E. aerogenes* are lactate, ethanol, acetate, succinate, and 2,3-butanediol.Unfortunately, because of the complexity of cellular metabolism, it was hard to describe the interaction between the formate pathway and the NADH pathway for the hydrogen production in a quantitative way. The two substrates, formate and NADH, are both important metabolites in the complicated cellular metabolic network. It might be more feasible to understand the hydrogen metabolism not only from the local point of view but also from the global point of view.

In essence, biohydrogen production is a routine of releasing excess electrons or energies in anaerobic metabolism. It is proposed that the global regulation of anaerobic metabolism might alter the electron or energy flow to affect the hydrogen yield. Our findings demonstrated that the formate pathway and the NADH pathway might interact with each other indirectly through the global regulation. The global regulation of anaerobic metabolism redistributed the hydrogen metabolism into the formate pathway and the NADH pathway. However, how to redistribute is still unclear and will need further investigation.

Since global regulation of anaerobic metabolism on the reducing power or energy level can alter the hydrogen production, the hydrogen yield may have tight interactions with cellular reducing power or energy level. Intracellular reducing power is typically stored in the form of NADH. The major cellular energy currency molecule is ATP. This study quantitatively showed the relationships of hydrogen production with cellular reducing power and energy level. The important roles of cellular NADH and ATP in hydrogen metabolism have been demonstrated, thus providing a basis for deciphering the essence of the hydrogen production. Based on this finding, metabolic regulations could be better rationalized to allow for improving biohydrogen production.

In conclusion, this work has presented how the perturbation of the formate pathway and NADH pathway affected the biohydrogen production. To enhance the hydrogen yields, many genetic engineering attempts have been focusing on two dominant metabolic pathways: formate pathway and NADH pathway. These two pathways have been studied independently, but this perturbation study exhibited that these two pathways interact with each other indirectly through the global regulation of reducing power and energy level. There are linear relationships between the relative change of hydrogen yield and the relative change of NADH yield or ATP yield. To further improve the hydrogen yield, an effective strategy would be to introduce intracellular NADH or ATP regeneration pathways to improve the NADH or ATP yields. This finding delineates a rationale for improving the hydrogen productivity from a global point of view that may contribute to our understanding of hydrogen metabolism.

## Methods

### Strains and plasmids

*E. aerogenes* IAM1183 was purchased from the Institute of Applied Microorganisms of the University of Tokyo, Japan. A list of bacterial strains, mutants, plasmids and recombinant plasmids used in the study are shown in Supplementary Table S1. The genes *hycA*, *ldhA*, *nuoB*, and *hybO* in *E. aerogenes* IAM1183 were knocked out, forming the mutant *E. aerogenes*-*ΔhycA*, *E. aerogenes*-*ΔldhA*, *E. aerogenes*-*ΔnuoB*, and *E. aerogenes*-*ΔhybO* ^30^. The genes *fdhF*, *fhlA*, *fdh1*, *narP*, and *ppk* were inserted into the multiple cloning sites of the plasmid pMCL to form recombinant plasmids, including pMCL-*fdhF*, pMCL-*fhlA*, pMCL-*narP*, pMCL-*fdh1*, and pMCL-*ppk* ^6^. The genetic analysis has been performed in previous studies (Supplementary Table S1). The recombinant plasmids were chemically transformed into *E. aerogenes* strains, and the empty plasmid pMCL was used as the control.

### Medium preparation

The culture medium (1 liter) consisted of glucose 15.0 g, tryptone 5.0 g, (NH_4_)_2_SO_4_ 2.0 g, KH_2_PO_4_ 14.0 g, K_2_HPO_4_.3H_2_O 6.0 g, and MgSO_4_ 0.2 g^6^. All chemicals were of analytical grade. The glucose was sterilized individually by autoclave. The initial pH value of the medium was controlled at 6.0 by the addition of NaOH or HCl.

### Cell cultivation

Twenty mL of medium was put into the serum bottle with a total volume of 70 mL. The serum bottle was air-sealed with a butyl rubber stopper and degassed with 100% of aseptic nitrogen for 5 minutes. The cultivation was performed at 37 °C on a 170 rpm shaker for 15 h. When the bacterial cells cultivated in a 100 mL Erlenmeyer flask containing 20 mL medium reached 1.0 in terms of optical density at 660 nm (OD_660_), 1 mL of the culture as the inoculum was transferred into the serum bottle. The whole operation was done under anaerobic conditions. The plasmids were induced by 0.5 mM IPTG. The inducers were added into the culture at the beginning.

### Analysis of cell density and glucose concentration

Cell densities at 600 nm (OD_600_) were measured with an ultraviolet-visible spectrophotometer (UV757CRT, Shanghai Precision & Scientific Instrument CO., Ltd., China). The dry cell weight of *E. aerogenes* was then calculated by the equation of dry cell weight (g/L) = 0.132 × OD_600_. The glucose concentration was measured by 3,5-dinitrosalicylic acid (DNS) colorimetry^32^.

### Measurement of hydrogen gas

Hydrogen was analyzed by a gas chromatograph (GC112A, Shanghai Precision & Scientific Instrument Co., Ltd., China) equipped with a thermal conductivity detector (TCD) and a 2 m × 3 mm (i.d.) stainless-steel column packed with TDX-01 (80~100 mesh). The temperatures of the injector, detector, and column were kept at 120, 120, and 80 °C, respectively. Nitrogen was used as a carrier gas at a flow rate of 10 mL/min.

### Measurement of organic metabolites

The concentrations of organic acids and alcohols in metabolites were analyzed by high-performance liquid chromatography (HPLC) (Shimadzu 10 A) equipped with a refractive index detector and a Shimadzu SCR-102H column after pretreatment with a 0.45 μm membrane filter. Five mM HClO_4_ was used as a mobile phase at a flow rate of 1 mL/min. The retention times of succinate, lactate, formate, acetate, 2,3-butanediol, and ethanol were 6.681 min, 8.666 min, 9.404 min, 10.088 min, 11.746 min, and 13.670 min, respectively.

### Analysis of the hydrogen yields

The hydrogen yield produced by the formate pathway was calculated from the sum of final acetate and ethanol minus the residual formate (H_2_ yield produced by formate pathway = (acetate + ethanol-formate)/consumed glucose). The hydrogen yield produced by the NADH pathway was calculated from the sum of the pathways producing NADH minus those consuming NADH (H_2_ produced by NADH pathway = ((2 × glucose + CO_2_ + CO_2_ consumed by succinate production-2 × 2,3-butanediol)-(2 × ethanol + 2 × succinate + lactate + 2,3-butanediol + H_2_-CO_2_))/consumed glucose).

## Electronic supplementary material

Supplementary Info
