# Supplementary material for: Perturbation of formate pathway and NADH pathway acting on the biohydrogen production
Source: Sci Rep. 2017 Aug 29;7:9587. doi: 10.1038/s41598-017-10191-7 (PMC5575262; doi:10.1038/s41598-017-10191-7)
Supplement: Supplementary file 1 — Supplementary Info [file 41598_2017_10191_MOESM1_ESM.pdf]

Supplementary Information

Perturbation of formate pathway and NADH pathway acting on the biohydrogen production

Dong Liu [1], Yunze Sun [1], Yuhao Li [1], Yuan Lu [1,2,3,*]

[1] *Department of Chemical Engineering, Tsinghua University, Beijing 100084, China.*

[2] *Key Lab of Industrial Biocatalysis, Ministry of Education, Department of Chemical Engineering, Tsinghua University, Beijing 100084, China.*

[3] *Institute of Biochemical Engineering, Department of Chemical Engineering, Tsinghua University, Beijing 100084, China.*

[*] *Correspondence to Yuan Lu, Department of Chemical Engineering, Tsinghua University, Beijing 100084, China; Email address: yuanlu@tsinghua.edu.cn; telephone: +86-10-62780127.*

Supplementary Tables

Table S1: Strains and plasmids used in this study. The genetic analyses have been performed in previous studies[1-8].

Strain and plasmid	Relevant genotype	Source or reference
E. aerogenes IAM1183	Wild-type, *Amp*^{r}	IAM
E. aerogenes-△*hycA*	△*hycA*, *Amp*^{r}, *Km*^{r}	1
E. aerogenes-△*ldhA*	△*ldhA*, *Amp*^{r}, *Tet*^{r}	2
E. aerogenes-△*nuoB*	△*nuoB*, *Amp*^{r}, *Tet*^{r}	3
E. aerogenes-△*hybO*	△*hybO*, *Amp*^{r}, *Tet*^{r}	1
pMCL	7.0 kb, *malE*, *lacI*^{q}, *Cm*^{r}	4
pMCL-*fdhF*	9.3 kb, *malE*, *lacI*^{q}, *Cm*^{r}	5
pMCL-*fhlA*	9.2 kb, *malE*, *lacI*^{q}, *Cm*r	5
pMCL-*fdh1*	8.2 kb, *malE*, *lacI*^{q}, *Cm*r	6
pMCL-*narP*	7.7 kb, *malE*, *lacI*^{q}, *Cm*r	7
pMCL-*ppk*	9.1 kb, *malE*, *lacI*^{q}, *Cm*r	8

Table S2: Genes manipulated for perturbing hydrogen-producing formate pathway or NADH pathway. FHL refers to formate hydrogen lyase.

Genes	Function	Reference
	**Related to H_2-producing formate pathway**	
hycA	FHL repressive regulon	1
focA	Formate transmembrane protein	9
fhlA	FHL activator protein	5
modE	Secondary transcriptional activator of FHL regulon	9
fdhF	Formate dehydrogenase H (FDH-H)	5
selC	Encoding a tRNA for incorporating selenocysteine to FDH-H	9
	**Related to H_2-producing NADH pathway**	
ldhA	Lactate dehydrogenase (A chain)	10
frdBC	Fumarate reductase	11
nuoB	NADH dehydrogenase/NADH-quinone oxidoreductase	3
adh	Alcohol dehydrogenase	2
nadE	NAD^+ synthetase	12
hoxEFUYH	NAD-reducing hydrogenase	13

References

1 Zhao, H. X. *et al.* Cloning and knockout of formate hydrogen lyase and H-2-uptake hydrogenase genes in Enterobacter aerogenes for enhanced hydrogen production. *Int. J. Hydrog. Energy* **34**, 186-194, doi:10.1016/j.ijhydene.2008.10.025 (2009).

2 Zhao, H. X. *et al.* Disruption of lactate dehydrogenase and alcohol dehydrogenase for increased hydrogen production and its effect on metabolic flux in Enterobacter aerogenes. *Bioresour. Technol.* **194**, 99-107, doi:10.1016/j.biortech.2015.06.149 (2015).

3 Ma, K., Zhao, H. X., Zhang, C., Lu, Y. & Xing, X. H. Impairment of NADH dehydrogenase for increased hydrogen production and its effect on metabolic flux redistribution in wild strain and mutants of Enterobacter aerogenes. *Int. J. Hydrog. Energy* **37**, 15875-15885, doi:10.1016/j.ijhydene.2012.08.017 (2012).

4 Lu, Y., Lai, Q., Zhang, C., Zhao, H. & Xing, X.-H. Alteration of energy metabolism in Enterobacter aerogenes by external addition of pyrophosphates and overexpression of polyphosphate kinase for enhanced hydrogen production. *J. Chem. Technol. Biotechnol.* **87**, 996-1003, doi:10.1002/jctb.3715 (2012).

5 Lu, Y., Zhao, H., Zhang, C., Lai, Q. & Xing, X.-H. Perturbation of formate pathway for hydrogen production by expressions of formate hydrogen lyase and its transcriptional activator in wild Enterobacter aerogenes and its mutants. *Int. J. Hydrog. Energy* **34**, 5072-5079, doi:10.1016/j.ijhydene.2009.04.025 (2009).

6 Lu, Y. *et al.* Expression of NAD(+)-dependent formate dehydrogenase in Enterobacter aerogenes and its involvement in anaerobic metabolism and H-2 production. *Biotechnology Letters* **31**, 1525-1530, doi:10.1007/s10529-009-0036-

z (2009).

7 Lu, Y., Zhao, H. X., Zhang, C. & Xing, X. H. Insights into the global regulation of anaerobic metabolism for improved biohydrogen production. *Bioresour. Technol.* **200**, 35-41, doi:10.1016/j.biortech.2015.10.007 (2016).

8 Lu, Y., Lai, Q., Zhang, C., Zhao, H. & Xing, X.-H. Alteration of energy metabolism in Enterobacter aerogenes by external addition of pyrophosphates and overexpression of polyphosphate kinase for enhanced hydrogen production. *Journal of Chemical Technology and Biotechnolog* **87**, 996-1003, doi:10.1002/jctb.3715 (2012).

9 Fan, Z. M., Yuan, L. & Chatterjee, R. Increased Hydrogen Production by Genetic Engineering of Escherichia coli. *PLoS One* **4**, 8, doi:10.1371/journal.pone.0004432 (2009).

10 Lu, Y. *et al.* Alteration of hydrogen metabolism of ldh-deleted Enterobacter aerogenes by overexpression of NAD(+)-dependent formate dehydrogenase. *Appl. Microbiol. Biotechnol.* **86**, 255-262, doi:10.1007/s00253-009-2274-3 (2010).

11 Yoshida, A., Nishimura, T., Kawaguchi, H., Inui, M. & Yukawa, H. Enhanced hydrogen production from glucose using ldh- and frd-inactivated Escherichia coli strains. *Appl. Microbiol. Biotechnol.* **73**, 67-72, doi:10.1007/s00253-006-0456-9 (2006).

12 Wang, J., Yu, W. Y., Xu, L., Wang, S. Y. & Yan, Y. J. Effects of increasing the NAD(H) pool on hydrogen production and metabolic flux distribution in Enterobacter aerogenes mutants. *Int. J. Hydrog. Energy* **38**, 13204-13215, doi:10.1016/j.ijhydene.2013.07.121 (2013).

13 Zheng, H., Zhang, C., Lu, Y., Jiang, P.-X. & Xing, X.-H. Alteration of anaerobic

metabolism in Escherichia coli for enhanced hydrogen production by heterologous expression of hydrogenase genes originating from Synechocystis sp. *Biochemical Engineering Journal* **60**, 81-86, doi:10.1016/j.bej.2011.10.006 (2012).
